# Aptamers: A Cutting-Edge Approach for Gram-Negative Bacterial Pathogen Identification

**DOI:** 10.3390/ijms25021257

**Published:** 2024-01-19

**Authors:** María Guadalupe Córdova-Espinoza, Rosa González-Vázquez, Rolando Rafik Barron-Fattel, Raquel Gónzalez-Vázquez, Marco Antonio Vargas-Hernández, Exsal Manuel Albores-Méndez, Ana Laura Esquivel-Campos, Felipe Mendoza-Pérez, Lino Mayorga-Reyes, María Angélica Gutiérrez-Nava, Karen Medina-Quero, Alejandro Escamilla-Gutiérrez

**Affiliations:** 1 Immunology Laboratory, Escuela Militar de Graduados de Sanidad, SEDENA, Mexico City 11200, Mexico; kmq.kmq5@gmail.com; 2 National School of Biological Sciences, National Polytechnic Institute, Laboratory of Medical Bacteriology, Mexico City 11350, Mexico; rosagonzvazq@yahoo.com.mx (R.G.-V.); rolandorafik@gmail.com (R.R.B.-F.); 3 Mexican Social Security Institute, Unidad Medica de Alta Especialidad, Hospital de Especialidades, “Dr. Antonio Fraga Mouret”, National Medical Center La Raza, Mexico City 02990, Mexico; 4 Laboratory of Biotechnology, Department of Biological Systems, Metropolitana Campus Xochimilco, CONAHCYT—Universidad Autonoma, Calzada del Hueso 1100, Col. Villa Quietud, Alcaldia Coyoacan, Mexico City 04960, Mexico; rgonzalezv@correo.xoc.uam.mx; 5 Research Department, Escuela Militar de Graduados de Sanidad, SEDENA, Mexico City 11200, Mexico; mavh78@yahoo.com.mx (M.A.V.-H.); albores_09@hotmail.com (E.M.A.-M.); 6 Laboratory of Biotechnology, Department of Biological Systems, Universidad Autonoma Metropolitana, Campus Xochimilco, Calzada del Hueso 1100, Col. Villa Quietud, Alcaldia Coyoacan, Mexico City 04960, Mexico; aesquivel@correo.xoc.uam.mx (A.L.E.-C.); fmendoza@correo.xoc.uam.mx (F.M.-P.); lmayorga@correo.xoc.uam.mx (L.M.-R.); 7 Laboratory of Microbial Ecology, Department of Biological Systems, Universidad Autonoma Metropolitana, Campus Xochimilco, Calzada del Hueso 1100, Col. Villa Quietud, Coyoacan, Mexico City 04960, Mexico; agutz@correo.xoc.uam.mx; 8 Mexican Social Security Institute, Unidad Medica de Alta Especialidad, Microbiology Laboratory, Hospital General “Dr. Gaudencio González Garza”, National Medical Center La Raza, Mexico City 02990, Mexico

**Keywords:** pathogens, aptamers, public health, pathogen identification, Kd, SELEX

## Abstract

Early and accurate diagnoses of pathogenic microorganisms is essential to correctly identify diseases, treating infections, and tracking disease outbreaks associated with microbial infections, to develop precautionary measures that allow a fast and effective response in epidemics and pandemics, thus improving public health. Aptamers are a class of synthetic nucleic acid molecules with the potential to be used for medical purposes, since they can be directed towards any target molecule. Currently, the use of aptamers has increased because they are a useful tool in the detection of specific targets. We present a brief review of the use of aptamers to detect and identify bacteria or even some toxins with clinical importance. This work describes the advances in the technology of aptamers, with the purpose of providing knowledge to develop new aptamers for diagnoses and treatment of different diseases caused by infectious microorganisms.

## 1. Introduction

Ninety-five years since penicillin was discovered [1], and despite the technological advances of our era, continued efforts are still being made to improve health systems worldwide due to emerging pathogen epidemics and the burden of hospital-care-associated infections (HCAIs), which today are a major public health concern globally [2,3]. In addition, the emergence of virulent and high-risk bacterial strains, such as “ESKAPE” pathogens (*Enterococcus faecium*, *Staphylococcus aureus*, *Klebsiella pneumoniae*, *Acinetobacter baumannii*, *Pseudomonas aeruginosa* and *Enterobacter* species), represent a global threat to human health [4]. Therefore, the rapid detection, quantification, and adequate treatment of infectious microorganisms are challenges to protecting public health [5]. Traditional methods for the detection of pathogenic bacteria are culture-based methods and biochemical tests, which are low cost, easy to operate, and highly standardized, but they lack differentiation between the target and other non-target endogenous microorganisms, they produce false negative/positive results, they are time- and labor-consuming procedures, and they are unable to detect viable but nonculturable cells [6]. Antibodies have made tremendous contributions in a wide range of applications. However, there are certain limitations associated with their use; monoclonal antibodies generally are incapable of membrane penetration due to their larger size and hence are less ideal as carriers for the targeted delivery of cytotoxic molecules inside cells. The production of monoclonal antibodies is laborious, expensive, time consuming, and suffers from batch-to-batch variations; they are also immunogenic, temperature sensitive, and their target binding kinetics cannot be easily modified [7]. In addition, detection with antibodies is not accurate when there are minimal amounts of microorganisms. In some cases, the detection is not precise due to the null specificity of some antibodies or due to false positives [8]. Thus, aptamers are considered to be an alternative to antibodies in many biological applications [9]. Molecular approaches such as quantitative PCR are often used for the rapid and accurate enumeration of pathogen-derived nucleic acids; however, some nucleases may inhibit the enzymes of PCR resulting in false-negative results [10]. Aptamers are proposed as substitutes for traditional detection methods; they include having an oligonucleotide single-stranded DNA (ssDNA) or RNA with target-selective high-affinity features, considered as nucleic acid-based affinity ligands [11,12], and which do not differ in specificity or affinity. RNA aptamers have greater flexibility and produce a greater variety of possible structural configurations [13]. Aptamers also have high stability, rapid production (synthesis or modification), low immunogenicity, are economic—they can be used for a wide range of targets—there are no animals needed for their production, they can be created against targets such as toxic or non-immunogenic molecules, and no cold chain for transportation and maintenance is needed [14]. DNA or RNA aptamers in comparison to antibodies can undergo reversible folding and unfolding, leading to a greater stability and a simpler elution of the bound target from the aptamer [15]; additionally, aptamers bind to their targets with high affinity and specificity due to their three-dimensional structure [12]. The binding of aptamers to their target results from the structure’s compatibility, electrostatic interactions, Van der Waals forces, hydrogen bonds, or a combination of these [16]. The affinity of the aptamers for their target molecule is measured using their dissociation constant (Kd) [17]. In the treatment of microbial infections, the aptamer-based systems have been found to be talented tools, regarding their promising anti-biofilm and antimicrobial activities. Aptamers can reduce or inhibit the effects of bacterial toxins, inhibit pathogen invasion to immune cells, and they can also be used in drug delivery systems [18]. To design aptamers, SELEX (Systematic Evolution of Ligands by Exponential Enrichment) has been employed as a useful technique for selecting nucleic acid ligands that interact with the target molecule in a desirable manner. SELEX is an iterative process of selection and amplification, in which large pools of nucleic acid molecules (>1 trillion distinct sequences) are challenged to bind to a desired target under defined conditions such as temperature and salt concentration; later, these sequences are amplified to generate a new population of enriched molecules. In SELEX, a negative selection is completed, which involves the incubation of a cell or microorganism closely related to the nucleotide sequences previously selected and the removal of sequences that are non-specific to the target molecule [19]. Finally, using bioinformatics programs, with secondary structures from the sequences selected, Kd, the different probable binding sites, and the structure of tertiary bonding are determined [20]. 

Due to all these characteristics, aptamers have been used as therapeutic or diagnostic tools, in analytical chemistry applications, as immobilized ligands, or in homogeneous assays, for the development of new drugs, and they have been used as aptasensors (biosensors that are based on aptamers as recognition elements), which can be constructed through a variety of methodologies, including electrochemical biosensors, optical biosensors, and mass-sensitive biosensors [9,21]. Sandwich-type biosensors, using a pair of aptamers, have also been reported; however, they are rarely available [22]. Here, we present a brief review of the use of aptamers for detecting and identifying bacteria or even some toxins with medical importance, since rapid detection and identification of the causal pathogen of the disease is crucial for public health; therefore, this review aims to provide information that can improve the diagnosis and treatment of various diseases caused by different pathogens.

## 2. Aptamers for Gram-Negative Bacteria Detection

Aptamers have become a topic of interest in clinical and food applications, since they either possess all the benefits of antibodies or special qualities, including heat stability, low cost, and limitless uses [23]. Table 1 shows several proposals about aptamer applications for detecting Gram-negative bacteria or some of their toxins with medical and food importance. Their unique features make aptamers a prominent tool in therapeutics, diagnostics, biosensors, and targeted drug delivery [23]. Thus, aptamers represent exciting materials for a variety of clinical and food applications, and can be modified to improve their properties and to extend their applications in human benefits.

### 2.1. Selection and Characterization of DNA Aptamers to Detect Acinetobacter baumannii

Rapid detection of *A. baumannii* is critical for limiting healthcare-associated infections and providing the best treatment for infected individuals. Su et al. [24] designed a dual-aptamer assay for the detection of *A. baumannii* on an electromagnetically driven microfluidic platform. The authors screened the AB-specific aptamer (5′-GACCACATATCACATGCTGTCGCCTTGCGA 0-ACAGCACCACA TATCAATTCCAGTGATGTTTGTCTTCCTGCC-3′) via SELEX with a Kd of 6.8 ± 1.9 nM. The automatic point-of-care device demonstrated the performance of a dual-aptamer assay to diagnose *A. baumannii* using an electromagnetically driven microfluidic system. Thus, this system will allow rapid clinical diagnoses of *A. baumannii* [24].

Park et al. [25] showed that a Lys AB2 P3-His–a hexahistidine-tagged form of an antimicrobial peptide (AMP) loaded onto DNA aptamer-functionalized gold nanoparticles (AuNP-Apt) can effectively inhibit *A. baumannii* infection in mice. The aptamer sequence consisted of 5′-GCTATGGGTGGTCTGGTTGGGATTGGCCCCGGGAGCTGGC-A10-f Thiol-3′. When *A. baumannii*-infected mice were intraperitoneally injected with AuNP-Apt loaded with Lys AB2 P3-His, a marked reduction in *A. baumannii* colonization was observed in the mouse organs, leading to a prominently increased survival time of the mice compared to the control mice. The authors suggested that AMPs loaded onto AuNP-Apt could be an effective therapeutic tool against infections caused by multidrug-resistant pathogenic bacteria in humans.

Farrel Côrtes et al. [26] designed a DNA aptamer for *A. baumannii* using in-house cell SELEX methodology. The sequence of A01 flanked by the APT-primers was 5′-CAGGGGACGCACCAAGGTTTTGTTTTTTCTTTGCTTCTTTTTGCTTTTTTTT CCATGACCCGCGTGCTGCGTGA-3′. The aptamer A01 demonstrated a binding preference to *A. baumannii* when compared to *K. pneumoniae*, *C. albicans*, and *S. aureus* in fluorescence assays. As the authors suggested, although the time-kill assay did not show an effect on bacterial growth, the bactericidal or bacteriostatic potential could not be totally discarded. 

### 2.2. Selection and Characterization of DNA Aptamers to Detect Campylobacter jejuni

*Campylobacter* species are etiological agents of campylobacteriosis, which is a significant foodborne disease worldwide. *C. jejuni* causes diarrheal diseases two to seven times more frequently than *Salmonella* spp., *Shigella* spp., or *E. coli* O157: H7. The most common sources of infection are contaminated food (raw or partially cooked meat, raw milk, contaminated water, seafood, chicken, and other poultry products, etc.) [61,62,63]. The Center for Disease Control and Prevention estimates that approximately 15% of campylobacteriosis cases lead to hospitalization. Traditional microbiological culture methods have evolved including selective media and the optimization of growth conditions for *C. jejuni*; however, an elevated incubation temperature (42 °C), microaerophilic conditions, and some resistance to antibiotics limit isolation, while for the latter the identification of *C. jejuni* includes phenotypic differentiation such as biotyping, serotyping, and multilocus enzyme electrophoresis [62,63]. Thus, it is of significant concern to develop rapid and reliable detection methods that enable real-time decisions.

Currently, antibodies are the most common ligands used for capturing and detecting a variety of bacterial pathogens [64]. However, the drawbacks of them are their complex preparation, differences in batches, susceptibility to temperature, and limited design flexibility which restrict their suitability for practical applications; therefore, the use of antibodies does not satisfy the increasing needs of the public. As a promising alternative, aptamers are an emerging class of biomacromolecules that rival antibodies in diagnostics [65]. To identify specific ssDNA aptamers against *C. jejuni*, Dwivedi et al. [27] used a cell-SELEX method. Binding affinity was determined using flow cytometry (n = 200,000); the percentage of fluorescent cells for the different aptamers ranged from 19% to 32%, using approximately 300 pmol of the aptamer sequence with 10^8^–10^9^ bacteria. To obtain the specific aptamer, a counterselection was performed with *B. cereus*, *E. coli* O157: H7, and *L. monocytogenes* ATCC-19115. The results obtained were of a few labeled cells, and were 4.71% for *B. cereus*, 1.26% for *E. coli* O157: H7, and 1.24% for *L. monocytogenes* ATCC-19115, compared to 51.72% for *C. jejuni* A9a, which indicated that the aptamer is highly specific to *C. jejuni* A9a. The ONS-23 aptamer had the highest affinity (31.44%) to *C. jejuni* A9a, with a sequence of 40 random nucleotides flanked by known sequences at the ends, and an outer-loop structure with 30 bases and three closure helices. The ONS-23 aptamer had a Kd of 292.8 ± 53.1 nM, indicating the high affinity presented against *C. jejuni*. 

Dehghani et al. [28] described a method for the colorimetric determination of *C. jejuni* in milk samples. It was based on the interaction of a specific DNA aptamer (5′-GCA AGATCT CCG AGA TATCGTGCTGGGGGGTGGTTTGTTTGGGTCGGT TGTTTTGGTTGGGCTGCAGGTAATACGTATACT-3′) with the surface protein in the cell membranes of *C. jejuni*. When the aptamer was bound by the target cells, the surface of the Au@Pd nanoparticles (NPs) were not blocked by the aptamer and the NPs exert a strong peroxidase-like activity. As little as 100 CFU/mL of *C. jejuni* can be detected in milk.

Kim et al. [29], using a highly specific aptamer ONS-23TA (5′-ACAAGGGACAGTAGACCAACAGGAAATCAAAGGCCGTGGGAA-3′) which binds to live *C. jejuni* and *E. coli*, improved an aptasensor with high effectivity for testing pure culture samples. The accuracy of the newly developed platform was comparable (p¼0.688) to that of the gold-standard detection method of the tazobactam supplemented culture. However, it was superior to the official agar-based detection method (p¼0.016) in a validation study with 50 naturally contaminated chicken carcass samples. This provided the first evidence that both morphological status and the amount of *Campylobacter* present play key roles in the effectiveness of colorimetric detection.

### 2.3. Selection and Characterization of DNA Aptamers to Detect Escherichia coli

Contamination by food or water microorganisms is a health and public safety problem. Therefore, detecting and identifying pathogens is essential for clinical diagnoses, water and environmental analyses, and food safety. *E. coli* O157: H7 is one of the most important serotypes associated with food-poisoning outbreaks. Over 700 strains or serotypes of *E. coli* are present in nature, water, and foods, several of them with virulence factors encoded mainly on mobile genetic elements, plasmids, or pathogenicity islands. Methods for rapid detection of *E. coli* and its related toxins are key to minimizing the risk related to foodborne pathogens. Consequently, a lot of methods are being developed to detect this bacterium in water and foods, with the microbial culture, bacterial count, and biochemical test considered as the gold standard. However, these methods are labor intensive, slow, and time consuming (2–10 days for confirmation). Therefore, novel, accurate, fast, and low-cost detection methods are needed [66,67].

In the study by Kim et al. [68], ssDNA aptamers were selected and characterized to identify *E. coli*. Using cell SELEX, a total of 28 ssDNA aptamers were obtained. Four ssDNA aptamers (E1, E2, E10 and E12) showed high affinity and selectivity for *E. coli*. Aptamer E1 (5′-ACTTAGGTCGAGGTTAGTTTGTCTTGCTGGCGCATCCACTGAGCG-3′), with a Kd of 12.4 nM; aptamer E2 (5′-CCATGAGTGTTGTGAAATGTTGGGACACTAGGT GGCATAGAGCCG-3′), with a Kd of 25.2 nM; aptamer E10 (5′-GTTGCACTGTGCGG CCGAGCTGCCCCCTGGTTT GTGAATACCCTGGG-3′), with a Kd of 14.2 nM; and the aptamer E12 (5′-GCGAGGGCCAACGGTGGTTACGTCGCTACGGCGCTACTGGTTGAT-3′), with a Kd of 16.8 nM. The measure was made using fluorescence to estimate the binding affinity of the ssDNA aptamers to the bacteria (10^7^). All ssDNA aptamers had a stem and loop structure, with the E2 aptamer having the highest binding affinity. These aptamers can be used for rapid and economical surveillance of microbial contamination with *E. coli* in water or food samples. Therefore, these aptamers can be used as probes in *E. coli* biosensors and as an alternative to antibodies to identify the microorganism.

An aptamer for the identification of *E. coli* O157:H7 has been reported by Yu et al. [30] with the selected aptamer found using SELEX and is specifically bound to *E.* coli O157:H7. The aptamer was applied to a quartz crystal microbalance (QCM) sensor for rapid and sensitive detection of target bacteria. A total of 19 rounds of selection against live *E. coli* O157:H7 and 6 rounds of counter selection against a mixture of other pathogens were performed. The aptamer pool from the last round was cloned and sequenced. One sequence S1 that appeared 16 times was characterized and showed a Kd of 10.30 nM. Subsequently, a QCM aptasensor was developed for the rapid detection of *E. coli* O157:H7. The limit of detection (LOD) and the detection time of the aptasensor was 1.46 × 10^3^ CFU/mL and 50 min.

Bruno et al. [31] designed EcO 4R, which is an aptamer for the detection of *E. coli* 8739. The degenerate SELEX template library sequence was 5′-ATCCGTCACACCTGCTCT-N36-TGGTGTTGGCTC CCGTAT-3′, where N36 represents the randomized 36-base region of the DNA library. Forward and reverse primer sequences were 5′-ATACGGGAGCCAACACCA-3′ and 5′-ATCCGTCACACCTGCTCT-3′ (designated reverse). The system allowed the detection of as few as 30 live unlabeled *E. coli* per mL. 

Savory et al. [39] designed an aptamer, selected using SELEX, designated as EcA5-27, which was shown to have high affinity and specificity for target cells, and the potential to discriminate between distinct strains of *E. coli*, thus with potential to detect inter-strain differences in *E. coli* including the NSM59 strain, (used as the target strain for aptamer selection) and to selectively identify uropathogenic isolates. The author suggested that the aptamer has significant potential value in the development of rapid point-of-care diagnostic tests, as well as in the fundamental understanding of bacterial physiology and pathogenesis.

### 2.4. Selection and Characterization of DNA Aptamers to Detect Francisella tularensis

*F. tularensis* is a small bacillus, Gram-negative, capsulated, and non-mobile bacteria, which causes tularemia. It is a severe lung disease in humans and other mammals, including squirrels, cats, and prairie dogs. Symptoms of tularemia include rapid development of fever with shaking chills, fatigue, and headaches. Humans acquire tularemia through contact with infected animals. The most prevalent subspecies is type A (*tularensis*). Interest in *F. tularensis* has recently increased due to its potential use as a biological warfare agent [33]. 

The classical method for identification of *F. tularensis* is the isolation of the pathogen through cultivation with subsequent identification using agglutination or immunofluorescence assay [69]. This method is time consuming and requires intensive handling of the infectious agent. Antigen detection in urine and RNA hybridization of wound specimens have also been used as diagnostic methods [27,70]. There are other methods such as PCR, capture-enzyme-linked immunosorbent assay (cELISA), or MALDI-TOF MS that could be used in emergent situations. However, faster and more feasible methods are still required [71].

Vivekananda et al. [34] selected 25 ssDNA sequences with high specificity to *F. tularensis* subspecies *japonica*. These were tested using Aptamer-Linked Immobilized Sorbent Assay (ALISA), and, subsequently, a dot blot analysis was completed. Aptamer-selected sequences showed specificity only for the bacterial surface antigen of *F. tularensis* (SCHU4 by SELEX) but not for *B. henselae* (negative control), thereby exhibiting specificity in its ability to bind only to the tularemia bacterial antigen from subspecies *japonica*, *holarctica* (also known as *palaearctica*) and tularensis but not to other bacteria such as *B. henselae* and to pure chicken albumin or chicken lysozyme.

Thus, the authors suggested that the mix appears to be a novel antitularemia aptamer cocktail with a possible application as a detection reagent. 

### 2.5. Selection and Characterization of DNA Aptamers to Detect K. pneumoniae

Chen et al. [36] reported a novel method of combining aptamer Ag_10_NPs based microfluidic biochips with bright field imaging for the detection of KPC-2-expressing bacteria; the resulting aptamer was rapid, simple, and specific for *K. pneumoniae* detection. The biochip was combined with bright field imaging, and then the captured bacteria could be observed and counted directly without using extra chemical labeling. KPC-2-expressing *E. coli* was used as the target bacterium with a detected limit of 10^2^ CFU and more than a 90% capture efficiency. The author suggested that the implemented method is remarkably specific towards KPC-2 *E. coli* over other non-resistant bacteria, and pathogen assay, which in fact only takes ~1 h to complete in a ready-to-use microfluidic biochip. Furthermore, the effective capture and fast counting of the microfluidic biochip system demonstrates its potential for the rapid detection of antibiotic-resistant bacteria.

Deb et al. [37] described a genetic approach for the fabrication of a prototype to the noninvasive detection of *K. pneumoniae* MTCC-7028 using a tailor-made plasmonic aptamer–gold nanoparticle (AuNP)assay. The sequence of the aptamer KPBA1 was 5′-/5ThioMC6-D/GGCTGGATGGGGCGTGTGGAGCCCCGTTAGAATATCAGAGGTGGTGGCAACGGTGCGGACAGCG-3′. The authors reported the assay as advantageous because the adsorbed specific aptamer reduced and/or eliminated false-positive responses to nontarget analytes. The authors demonstrated the specific detection of *K. pneumoniae* bacteria with LOD of 3.4 × 10^3^ CFU/mL in 5 min, which is less than the time the current detection method takes (24 h).

### 2.6. Selection and Characterization of DNA Aptamers to Detect Neisseria meningitidis

Mirzakhani et al. [38] developed a DNA aptamer for screening of *N. meningitidis* serogroup B using SELEX. *N. meningitidis* is the causative agent of meningitis responsible for about 1% of infections in an epidemic period. In this study, the authors reported the selection of 60 clones, which bound efficiently to 21 aptamers; the binding was assessed using flow cytometry. The aptamers K3 and K4 showed the highest affinity to *N. meningitidis* serogroup B and no affinity to serogroups Y, A, C, or to other meningitis-causing bacteria. The Kd values for K3 and K4 were 28.3 ± 8.9 pM and 39.1 ± 8.6 pM, respectively. The K3 aptamer with the lowest Kd was chosen as the main aptamer, which allowed for the detection of *N. meningitidis* in cerebrospinal fluid (CSF) samples from patients and in CSF from healthy volunteers inoculated with *N. meningitidis* serogroup B (ATCC 13090) at 200 and 100 CFU/mL to samples of cerebrospinal fluid (CSF) from patients and samples of CSF from healthy volunteers inoculated with *N. meningitidis* serogroup B, respectively. 

### 2.7. Selection and Characterization of DNA Aptamers to Detect Proteus Mirabilis

*P. mirabilis* causes catheter-associated urinary tract infections (CAUTIs), and there are currently no effective means available to prevent *P. mirabilis* infections; strategies for diagnosis and prophylaxis are urgently needed. Therefore, aptamers offer a potential tool for diagnoses and as therapeutic agents against this bacterium. As a food pathogen, it can lead to food poisoning and even death in severe cases; food poisoning has been reported worldwide [72]. When intoxication occurs, the quantification and qualitative detection of *P. mirabilis* has a significant diagnosing criterion for treatment. Thus, it is greatly important to establish rapid detection, since culture methods for detection are time-consuming and inefficient [41].

Current identification of *P. mirabilis* is based on microbial characteristics, for example enzyme-linked immunosorbent assay (ELISA) or molecular biology techniques based on PCR assays, since accurate and rapid identification is necessary for early treatment. In this sense, immunochromatographic assays for antimicrobial detection are being used; both have some limitations, since they cannot directly detect the microorganism [41]. Due to these limitations, aptamers or aptamer-based biosensors are considered as another approach with a high potential for bacterial recognition [73,74].

In the work carried out by Savory et al. [39], the application of cell SELEX was demonstrated for the identification of ssDNA aptamers with high affinity for *P. mirabilis*, using a 66 nt ssDNA library with a 24 nt random region (N24) flanked by two known 18 nt sequences (5′-ATTACTTACGCTATCTAA(N24)TTATCATCTGGTATGTTA-3′). The aptamers PmA102 (5′-GGGGGGGGAACATGTTTGGGGGGG-3′) and PmA109 (5′-TT GCTTTAGGGGAGGTTGGGGGGG-3′) presented a high affinity in dot blotting assays. The specificity of aptamers was assessed using selection methods against *P. vulgaris*, *K. pneumoniae*, *A. baumannii*, *S. aureus*, *M. morganii,* and *E. coli* NSM59, which were immobilized on a nitrocellulose membrane and incubated with 600 pmoles of FITC-labeled aptamer (fluorescein isothiocyanate). To improve the specificity of the aptamers against *P. mirabilis,* an in silico maturation (ISM) was used. Two cycles of ISM (random recombination and point mutations) allowed the identification of an aptamer with a specificity greater than 36%. The affinity of aptamers was evaluated using a test in which a range of concentrations of PmA102 or PmA109 (0.5 to 200 nM) were incubated with *P. mirabilis* immobilized on a nitrocellulose membrane. PmA102 showed a Kd of 3.5 nM and a PmA109 a Kd of 7.7 nM for *P. mirabilis*. Thus, the aptamers described constitute the basis for developing novel biosensors and point-of-care devices which can facilitate an early diagnosis of infection and function as novel therapeutics. Furthermore, the selected aptamers can be useful tools for the study of membrane-associated proteins and the complex multicellular behaviors of this organism to block the biofilm formation. 

Elumalai et al. [40] demonstrated the antibiofilm activity, inhibition of swarming motility, and cell viability at a concentration of 3 μM of PmA2G02 aptamer targeting *P. mirabilis* 1429^T^. Also, PmA2G02 showed a binding affinity towards fimbria outer membrane usher protein (PMI1466), flagellin protein (PMI1619), and the regulator of swarming behavior (rsbA), which are responsible for adhesion, motility, and quorum sensing, respectively. The DNA aptamer sequence PmA2G02 was 5′-ATTACTTACGCTATCTAATTTTGCTG TAGGGGAGGAGGGTGGGTTTTTTATCATCTGGTATGTTA-3′. The aptamer was composed of 66 nucleotides and had a GC content of 38%. The melting temperature of the aptamer was 37 °C, indicating its stability under physiological conditions. It has a maximum of 50 possible folds and exhibits a maximum secondary-structure stability of 1.56 (ΔG).

Yao et al. [41] described an amino-modified aptamer (DNA sequence of NH2-TTGCTGTAGGGGAGGAGGGTGGGT) conjugated to the surfaces of SiC QDs (DNA-SiC QDs) (aptamer-modified Sic quantum dots), synthesized through one-pot hydrothermal method with particle sizes of about 14 nm, for *P. mirabilis* recognition. The authors reported that the union of aptamer-*P. mirabilis* causes a decrease in the fluorescence intensity of DNA-SiC QDs. The LOD was 526 CFU/mL (S/N =3). The aptasensor was used for the determination of *P. mirabilis* in pure milk samples and had good accuracy (87.6–104.5%), and recovery rates (85–110.2%) were obtained. The detection obtained in the simulated forensic identification samples (pure milk, milk powder, blood, and urine) gave satisfactory coincidence rates with the method of bacterial isolation and identification as standard. Thus, the authors concluded that the results demonstrated the potential of the fluorescent aptasensor for identification of *P. mirabilis* in forensic food-poisoning cases.

### 2.8. Selection and Characterization of DNA Aptamers to Detect Pseudomonas aeruginosa

*P. aeruginosa* is commonly found in the environment, particularly in fresh water. Infections with this pathogen are common in immunocompromised individuals from a variety of factors such as cancer, transplants, burns, neutropenia, AIDS, organ transplant, or cystic fibrosis. Also, it is among the most important causes of nosocomial infections due to its extreme versatility and antibiotic resistance [75].

Diagnoses of *P. aeruginosa* infections are usually based on cultures from blood, urine, or respiratory samples. Aside from vaccines and antibiotics, monoclonal antibodies (mAbs) have been deployed to control the spread of *P. aeruginosa* and lower infection severity. mAbs are viewed as a treatment option for high-risk individuals for whom vaccination is not an option; however, the use of mAbs is still expensive and not feasible for everybody. Although using therapeutic monoclonal antibodies is a promising method for identifying or restricting infections, some limitations are observed such as dose-dependent, passive immunization that often requires higher doses of antibodies against active bacterial infections [76].

To combat *P. aeruginosa* infections efficiently and specifically, proper methods for pathogen identification and antimicrobial-susceptibility testing (AST) are required [77], since most techniques require multiple steps including isolation, enrichment, and/or purification for increasing the assay’s time and cost. Thus, a potent new method is necessary to identify the bacteria accurately, rapidly, and simply, with aptamers being a feasible option [43].

In the study by Wang et al. [42], ssDNA aptamers were selected using cell SELEX. In addition, a rapid FISH (fluorescence in situ hybridization) method was used using the ssDNA aptamer as a test to detect *P. aeruginosa*. The ssDNA library consisted of a randomized central sequence of 60 nucleotides (nt) flanked by two sequences recognized using primer 5′-ATACCAGCTTATTCAATT-60nt-AGATAGTAAGTGCAATCT-3′. The selected aptamers were F17 (5′-CCCCAGTGCTCAGCTTCTCCTCCGTCTCATTCTTCTGTAGGATCGTTCTGTTTTGGTACT-3′) and F23 (5′-CCCCCGTTGCTTTCGCTTTTCCTTTCGCTTTTGTTCGTTTCGTCCCTGC TTCCTTTCTTG-3′). The Kd values of these aptamers were 17.27 ± 5.00 nM for aptamer F23 and 57.63 ± 11.64 nM for aptamer F17. The aptamer F23 showed a high affinity for *P. aeruginosa* but not for *A. baumannii* or *S. maltophilia* using flow cytometry. For the characterization of the binding affinity of aptamers F17 and F23, fluorescence intensity of the aptamers was determined. This study demonstrated the value of aptamer-FISH assays in bacterial detection, which can provide reliable, rapid, and economical detection of *P. aeruginosa* against other Gram-negative bacteria present in a mixed nosocomial infection. Furthermore, the whole procedure could be finished in 1.5–2 h.

In another study by Wang et al. [43], ssDNA aptamers were selected using cell SELEX, and the rapid FISH method was used as the binding validation method. The ssDNA library consisted of a randomized central sequence of 40 nucleotides (nt) flanked by two sequences recognized by primer 5′-TGGACCTTGCGATTGCGATTGA CAGC GCAGA CATGAGTCTCAGGAC-3′. Five aptamers were proved. The selected aptamer was PA1 (5′-TGGACCTTGCGATTGACAGCAGACATGAGTCTCAGGACGTGACCGCTGGACCTTGCGATTGACAGCAGACATGAGTCTCAGGAC-3′), ΔG–7.60 kcal/mL, and Kd 15.16 ± 3.62 nM, the rest of the proved aptamers were discarded due to their ΔG, Kd, and specificity, since PA1 exhibited significantly lower (*p* < 0.05) fluorescence-recovery ratios against the interfering bacterium tested (*P. putida*, *E. faecalis*, *C. perfringens*, *E. coli*, *B. subtilis*, and *S. aureus*). In this study, fluorescence assay exhibits high specificity and sensitivity. Moreover, the authors suggested that the assay was easy to operate and comprises environmentally friendly materials, has potential applications on actual water samples with satisfactory accuracy, and that these characteristics highlight that the assay has potential in the application of detecting the contamination of *P. aeruginosa* in water. 

Zhong et al. [44] determined *P. aeruginosa* using aptamer-modified magnetic nanoparticles and a fluorometric assay based on the hybridization of the aptamer (5′-CCCCCGTTGCTTTCGCTTTTCCTTTCGCTT TTGTTCGTTTCGTCCCTGCTTCCTTTCTTG-C6 NH2-3′) and fluorescein-labeled complementary DNA (FAM-cDNA) (5′-6-FAM-ACGAACAAAAGCGA-3′) in combination with magnetic separation. This assay allowed both the selective enrichment and sensitive fluorometric determination of bacteria in a single step. In the absence of *P. aeruginosa*, FAM-cDNA was assembled on the surface of aptamer-modified magnetic particles (MNPs) using the hybridization between the aptamer and cDNA. After the addition of *P. aeruginosa*, FAM-cDNA was replaced with the bacteria and released from the MNPs, since the aptamer preferentially binds to bacteria. The number of bacteria was determined using the fluorescence intensity (λexc/em = 494/525 nm) of the supernatant containing the released FAM-cDNA. The assay had a response in the range between 10 and 10^8^ CFU/mL of the tested bacteria, with an LOD as low as 1 CFU/mL. 

### 2.9. Selection and Characterization of DNA Aptamers to Detect Shigella spp. 

*Shigella* species are facultative intracellular Gram-negative pathogens that cause shigellosis, which remains a significant public health concern. *Shigella* has four species: subgroup A (*S. dysenteriae*), subgroup B (*S. flexneri*), subgroup C (*S. boydii*), and subgroup D (*Shigella sonnei*) [78]. This bacterium causes almost 165 million cases of diarrhea annually. The most common symptoms of shigellosis are light watery diarrhea, vomiting, fever, tenesmus, headache, and abdominal pain [79].

The illness is generally self-limiting but may become life threatening in patients with a compromised immune system or in the absence of adequate health care. Because of the low infectious dose of *Shigella* spp. (10–100 organisms) compared to other enteropathogens shigellosis is a serious public health threat [80]. 

Identification at species and serotype levels is a crucial task in microbiological laboratories. Nevertheless, the high similarity between *Shigella* spp. and *E. coli* is a great challenge with the subsequent negative repercussion on surveillance, epidemiological investigations, and selection of appropriate treatments [81]. Multiple techniques have been developed ranging from phenotype-based methods and single or multilocus molecular techniques to whole-genome sequencing (WGS), but they are time consuming or expensive. Aptamers became an option for differentiating between identification using a high sensibility and specificity alone and those using another technique such as an enzyme-linked sedimentation assay (ELASA) [80]. 

#### 2.9.1. Specific Detection of *Shigella sonnei* Using Enzyme-Linked Aptamer Sedimentation Assay

Consumption of food contaminated with infectious or toxicogenic microorganisms is a significant cause of morbidity and a major cause of death worldwide. Many cases of acute diarrheal diseases in both developed and industrialized countries are due to infection with species of *Shigella*. *S. sonnei* is the species with the highest incidence in developing countries, one of the most frequent in tropical areas, and transmission mainly through direct contact with infected individuals (fecal–oral route) [82].

Masoudipour et al. [45] used cell SELEX to select ssDNA aptamers able to recognize *S. sonnei*. The affinity and specificity of the aptamers were tested using ELASA. Five ssDNA sequences (ASA1, ASA2, ASA3, ASA4, and ASA5) were selected from a library of aptamers. The aptamer ASA4 had a higher affinity with *S. sonnei* (10^9^ CFU). This work suggest that the aptamer can be applied in different areas that are compromised by the presence of this pathogenic microorganism.

In another study by Gong et al. [46] the selection, identification, and application of dual DNA aptamers against *S. sonnei* was completed. A pair of DNA aptamers bound to *S. sonnei* were selected based on whole-cell SELEX and identified with flow cytometric analyses. The authors also developed a fluorescent-sandwich-type identification using dual aptamers with different secondary structures such as capturing and detecting probes separately. The aptamer showed an LOD of 30 CFU/mL with a high sensibility and specificity, indicating the advantages of its use over other methods of detection that will potentially contribute to the development of a biosensor for analytical systems detecting *S. sonnei*.

#### 2.9.2. In Vitro Selection of an ssDNA Aptamer Directed towards *Shigella dysenteriae*

Shigellosis is a contagious enteric infection caused by *Shigella*, with *S. dysenteriae* being the leading cause of bacillary dysentery. In a study by Duan et al. [47], they used the cell-SELEX method to select aptamers against this species. A group of 24 aptamers were cloned and sequenced. According to the homology of the ssDNA, sequences were classified into nine families, and the binding was chosen for each family based on the principle of free minimum energy. Aptamer SI (5′-CGGAACTAGCGTTTAAATGCCAGGACTGAAGTAGGCAGGG-3‘) showed the highest affinity to *S. dysenteriae.* The Kd and ΔG of S1 was 23.47 ± 2.48 nM and −2.7 kJmol^−1^.

To characterize the S1 aptamer, it was labeled with FAM, and a counter selection was performed with *S. boydii*, *S. flexneri*, *S. sonnei*, *S. enterica* serovar Typhimurium, and *E. coli*. This work demonstrated that the S1 aptamer and aptamer-based tests could represent new strategies to develop simple and sensitive detection methods against pathogenic bacteria for food quality and safety control to overcome the tedious isolation and purification requirements for complex targets. 

In a work by Bruno et al. [48], two aptamers were selected to discriminate between *S. boydii* and *S. dysenteriae* from very closely related *E. coli*. Aptamers were selected from a pool of 84 sequenced aptamers developed against each of the four subgroups of *Shigella*. 

### 2.10. Selection and Characterization of DNA Aptamers to Detect Salmonella spp.

Enteric fever is caused by *S. typhi* and *S. paratyphi* A and remains one of the major diseases of upmost public health importance, since they are human-restricted pathogens, transmitted through the fecal–oral route. Conventional culture methods for detecting bacterial pathogens require a long incubation time, expensive immunoassay methods, and sample-enrichment steps. The search for novel methods for detecting this bacterium to avoid outbreaks is crucial. Aptamers’ development could be an alternative due to their low detection range and high sensitivity and specificity; moreover, they can be used alone or coupled with other techniques for on-site detection [83].

#### 2.10.1. Detection of *Salmonella enterica* Serovar Typhimurium Using Cell SELEX

Compared to 31 pathogens, which are considered the most common causes of diseases associated with food, *Salmonella* is responsible for about one third of all cases of foodborne bacteria and the second leading cause of food borne transmitted diseases. *Salmonella enterica* serovar Typhimurium is one of the serotypes that has been frequently associated with human infections since 1997 [84]. Human salmonellosis is usually associated with the consumption of contaminated food mostly from animal sources such as poultry, eggs, milk, meat, vegetables, fruits, and non-animal-sourced spices. Dwivedi et al. [50] obtained several DNA aptamers that could bind specifically to the *S. enterica* serovar Typhimurium strain (S913) using cell SELEX and a sample of an infected person. They used a combined library of biotin-labeled ssDNA. The aptamers that bound *Salmonella* were sorted, cloned, sequenced, and characterized using their binding specificity. The aptamer S8-7 (5′-CTGATGTGTGGGTAGGTGTCGTTGATTT CTTCTGGTGGGG-3′) had a relatively higher affinity than the rest of the aptamers with a Kd of 1.73 ± 0.54 μM and a loop structure which consisted of forty-five bases and two propellers closing. This study demonstrated that biotin-labeled aptamers selected using cell SELEX can be employed in a qPCR to detect *S. Typhimurium*.

#### 2.10.2. Detection of *Salmonella* O8 

*Salmonella* infections remain a big problem worldwide, causing enteric diseases, with *S. typhi* or S. *paratyphi* being the main cause. However, non-typhoidal *Salmonella* (NTS) in healthy individuals has caused enteric fever in recent years. NTS may become invasive and cause septicemia in elderly or immune-compromised individuals, leading to high mortality and morbidity rates. As invasive NTS is restricted to several O-antigen serogroups including B1, D1, C1, and C2, O-antigen polysaccharide, they are believed to be a good target for vaccine development or as a strategy for targeting NTS such as *Salmonella* O8 [85,86].

In the study by Liu et al. [52], a rapid method for detecting *Salmonella* O8 was used with specific aptamers selected with a cell SELEX from a previously random library of 9 families of 78 bp ssDNA.

The aptamer affinity was determined using fluorescence, and the specificity was measured using ALISA assay, where the absorption intensity of *Salmonella* O8 was higher than the other control bacteria (*E. coli* O86: K61 and *S. choleraesuis*). Aptamer B10 was selected (5′-GATCCGGGCCTCATGTCGAACACCCCCCAACTAAAACAACAAAACACCACCGCCATTGAGCGTTTATTCTGAGCTCCCA-3′) and showed a high specificity and binding affinity with a dissociation constant of 32.04 nM.

This work provided a test for the detection of *Salmonella* O8 with an “in situ” assay, marking the aptamer with fluorescence, which can be a potential alternative for rapidly detecting foodborne pathogens.

#### 2.10.3. Detection of *Salmonella enterica* Serovar *paratyphi*

The rapid and accurate identification of *S. enterica* serovar Paratyphi A, from infected samples, is critical for the subsequent treatment of infectious diseases caused by this bacterium’s genus.

Yang et al. [87] selected ssDNA aptamers (10, 22, 45, and 60 nucleotides) using the cell-SELEX method with a high affinity and specificity for *S. enterica* serovar Paratyphi A. The clinical strain used for the selection was isolated from patients infected with *Salmonella* (*S. enterica* serovar Paratyphi A); four *Salmonella* serotypes were used for negative selection (*S. enteritidis*, *S. typhimurium*, *S. choleraesuis*, and *S. arizonae*). With a fluorescence assay, the selected aptamers were determined to have a high binding capacity and specificity to this pathogen. To detect this bacterium with high specificity at a low cost, a new useful detection method based on non-covalent self-assembly of single-walled carbon nanotubes (SWNT) was constructed. Bacteria could be quantified using chemiluminescence-intensity changes at 420 nm, and the LOD of the method was 10^3^ CFU/mL. The value of Kd for aptamer 10 (5′-GATGATGGACGTATATCGTCTCCC ATGAATTCAGTCGGACAGCG-3′) was 73 ± 9 nM, for aptamer 22 (5′-ATGGACGAA TATCGTCTCCCAGTGAATTCAGTCGGACAGCG-3′) was 47 ± 3 nM, for aptamer 45 (5′-ATGGACGAATATCGTCTCCCAGTGAATTCAGTCGGACAGC-3′) was 68 ± 6 nM, and for aptamer 60 (5′-CGCCCACCCATAATGGATCAGGGCGGGCACCACGATG-3′) was 56 ± 9 nM. The aptamer 22 was chosen due to its Kd of 47 ± 3 Nm. This work demonstrated the applicability of *Salmonella* aptamers and their potential use in its detection in food, clinical, and environmental samples.

### 2.11. Selection and Characterization of DNA Aptamers to Detect Vibrio spp. 

*V. parahaemolyticus*, *V. harveryi*, *V. alginolyticus*, and *V. vulnificus* are important pathogens in the aquaculture environment [88,89,90], causing huge losses to the aquaculture industry; they can also infect humans through food and water and become a serious threat to public safety. Traditional methods have been used for the detection of these pathogens, which include microbial-testing techniques; instrumental analyses methods such as real-time polymerase chain reaction, molecular biology techniques, and immunological detection methods such as enzyme-linked immunosorbent assay; enzyme-linked fluorescence analysis; time-resolved fluorescence immunoassay; and chemiluminescence immunoassay [91]. However, these methods have some specific limitations/disadvantages for *Vibrio* detection. Aptamers have been proposed as an alternative to these limitations [92], which could be successfully selected using SELEX prior to knowing the corresponding target molecules [57].

#### 2.11.1. Detection of *Vibrio parahaemolyticus*

*V. parahaemolyticus* is an important seafood-borne pathogen with a serious impact on human health [93]. Consumption of fresh fish and seafood causes acute gastroenteritis, and it can be associated with wound infections in humans worldwide [94]. Although culture-based biochemical identification is widely used and isolation of the bacteria is the gold standard, these methods require laborious steps and are time-consuming. Thus, novel sensitive and rapid detection methods have been developed such as polymerase chain reactions (PCRs) [95,96] and ELISA [97]; these methods are still restricted, since specialized equipment and qualified operators are needed. Rapid, simple, and sensitive methods for screening *V. parahaemolyticus*-contaminated foods are urgently needed to ensure food safety.

In a study by Duan et al. [55], with a cell-SELEX method, they selected ssDNA aptamers that bind specifically to the strain ATCC 17802. The purpose of using live bacterial cells is to avoid a priori purification of specific target molecules from cell surfaces; additionally, live bacteria can grow in suspension, allowing simple separation using centrifugation. Flow cytometry was used to identify the aptamer bounded with high affinity. Aptamers A3 and A3P showed higher binding affinity, with fluorescence values around 75%. The aptamers A1, A1P, A3, A3P, and A18P were incubated with *L. monocytogenes*, *E. coli*, *S. typhimurium*, *S. aureus*, *C. sakazakii,* and *S. pneumoniae* as the negative control. The aptamer A3P(5′-FAM-ATAGGAGTCACGACGACCAGAATCTAAAAATGGGCAAGAAACAGTACTCGTTGAGAACTTATGTGCGTCTACCTCTTGACTAAT-3′) showed a high binding affinity with a 76% affinity for *V. parahemolyticus* and low affinity for the other bacteria (4%). The Kd was 16.88 +- 1.92 nM. This work was the first to report on the use of whole-bacterium SELEX for selecting specific aptamers for *V. parahaemolyticus,* allowing detection even when it is applied to a complex sample matrix, such as food. Also, these sequences of aptamers can be linked to magnetic nanoparticles to capture and conserve the bacteria with a magnetic field, or they can be chemically modified and conjugated to sensitive detection probes, etc. 

#### 2.11.2. Detection of *Vibrio alginolyticus*

*V. alginolyticus* is a pathogenic bacterium widely distributed in ocean, coastal, and estuarine areas, and it poses a threat to public health due to its significant impact on morbidity and mortality [98,99]. Consumption of raw seafood contaminated with *V. alginolyticus* or even exposure to contaminated water could result in bacterial infections such as gastroenteritis, otitis media, and chronic diarrhea. Diseases are reported worldwide including in Europe, Asia, North America, and South America. Moreover, indiscriminate use of antibiotics can lead to an increase in bacterial antibiotic resistance, so rapid, sensitive, and accurate methods for detecting this bacterium are needed [98]. 

The conventional methods for detection of *V. alginolyticus* comprise culture-based traditional microbial methods including biochemical identification, selective cultivation, serotype identification, toxin detection, and a wide range of PCR methods. However, those methods are still complicated, time consuming, expensive, and require expertise and advanced laboratory systems. New methods for providing a simple and efficient way to detect the bacterium for accurate diagnoses and treatment are being developed [98,100]. 

In a study conducted by Tang et al. [58] using formaldehyde-inactivated bacteria, aptamers against this bacteria were selected from an 82 bp random library of ssDNA using cell SELEX. All sequences obtained were divided into nine families according to their homology, and some conserved sequences were found in each of the six families (GCACAAGAGGGA), suggesting that the conserved sequence could be important to form the secondary structure of the aptamers.

After 15 rounds of selection, the final group of aptamers was highly specific for *V. alginolyticus,* with a dissociation constant of 27.5 ± 9.2 nM. The specificity was determined by contacting the aptamers with other bacteria (*V. alginolyticus*, *E. tarda*, *A. hydrophila,* and *V. harveyi*) showing greater specificity towards *V. alginolyticus*. Qualitative detection of the inactive microorganism at low concentrations (100 cells/mL) was demonstrated using the family of aptamers with a higher affinity and PCR. Thus, the successful identification of aptamers that can be used in the selective detection of *V. alginolyticus* may be more useful in the detection of pathogenic microorganisms in aquatic environments.

In another study, Zhao et al. [59] developed a novel method for the detection of *V. alginolyticus* utilizing a specific aptamer for recognition and signal amplification via a hybridization chain reaction (HCR) and horseradish-peroxidase-conjugated streptavidin. The biotin–aptamer conjugate binds to streptavidin on horseradish-peroxidase-conjugated streptavidin (SA-HRP), which allows for a highly sensitive and specific detection method. The development of these methods allows the detection of a linear range from 10 to 10^7^ CFU/mL with an LOD of 3 CFU/mL, which has a high potential for being used to monitor *V. alginolyticus* in aquaculture environments. 

#### 2.11.3. Detection of *Vibrio vulnificus*

*V. vulnificus* is a Gram-negative pathogenic bacterium that is motile, curved, and halophilic, inhabits estuarine or marine coastal areas, is one of the most important marine pathogens, and is highly pathogenic to humans [60]. Yan et al. [60] identified a highly specific DNA aptamer for Vibrio vulnificus using SELEX coupled with asymmetric PCR. After 13 rounds of cross-selection, the authors identified a novel DNA aptamer (Vapt2), which was characterized in terms of Kd and LOD; they also suggest that their work is a framework for the rapid detection of pathogenic bacteria and water pollution.

## 3. Detection of Gram-Negative Pathogens Using RNA Aptamers

RNA aptamers are defined as RNA oligonucleotides that bind to a specific target with high affinity and specificity. Isolation of aptamers has been completed by SELEX. They function similarly to antibodies, have low immunogenicity compared to other macromolecules; compared to peptides and antibody, they are easier to synthesize. Furthermore, nucleic acids such as RNAs are generally considered to be more thermodynamically stable than peptides or antibodies. RNA aptamers can be further chemically modified, which has been found to greatly improve their stability and resistance to RNAse. RNA aptamers have therapeutic and diagnostic applications and have been isolated against mostly cell surface markers, but they also have applications as intra- and extra-cellular components of key signaling pathways [101].

RNA aptamers can directly bind to extracellular targets to inhibit, and in some cases activate their functions. They can deliver a variety of therapeutic agents such as small molecules, peptides, and especially RNA-based therapeutics into specific cell types for the treatment of human diseases such as diabetes and cancer [101]. To perform early diagnostic assays and adequate therapy of infectious diseases, they can recognize pathogenic bacteria as well as block their functions [102], for instance, in the detection of *Salmonella enteric* using the RNA aptamer S-PS8.4, which specifically binds to type IVB pili of *S. enterica* serovar Typhi [103]. Another example is the 20-F-RNA aptamer I-1 against the *E. coli* O157:H7 strain, which was obtained using whole bacterial cells as the target [104]. On the other hand, 2′-F-RNA aptamers have been designed to detect *Pseudomonas aeruginosa* [105].

## 4. Conclusions

Aptamers are a promising new class of agents in the detection and treatment of pathogenic bacteria due to their different advantages over antibodies. During the last few years, there has been an increase in the number of experiments describing the identification of pathogenic bacteria using aptamers. SELEX allows for obtaining aptamers with high affinity to the target cell. Therefore, the number of rounds attributed to the aptamer in SELEX and the reagents that allow the union to reach their specific targets is essential and sometimes specific for each microorganism.

Aptamers can be developed against a whole bacterium, a microbial toxin, or some proteins to detect bacteria and diagnose disease. Those aptamers can be used alone or through combining various methods to potentialize the detection speed, convenience, cost effectiveness, and simplicity. Aptamers can be directed against (1) surface components, host cell receptors to block host cell entry or deliver drugs, (2) necessary proteins or enzymes to hamper bacterial pathogen propagation, (3) microbial toxins to relieve symptoms caused by toxins, or (4) pathogenic immunosuppressors or host immune-related molecules to augment host immune system functions. Since aptamers against the same pathogen have been developed, it is necessary to probe aptamer cocktails for infectious diseases [106].

The selection of aptamers against pathogens has great potential for diagnosing, investigating, and treating some diseases caused by pathogenic bacteria. Experiments using aptamers in the detection of pathogens can improve and even replace the tests commonly used to detect pathogens in the clinical area. It is expected that, in the following years, SELEX and its derivatives will obtain improvements that will help acquire and select a more significant and better quantity of aptamers, which would mean improved detection of pathogens causing various diseases. 

Numerous aptamers have been developed against various pathogens. Sequence information, binding affinity, development conditions for SELEX, and detection limits of these aptamers are needed for present and future investigations. Most of the described aptamers are selected for pathogens or to treat infectious diseases. Current information indicates that aptamer-based diagnoses and treatment platforms for communicable diseases are feasible and worth future research development [106].

In this sense, variations such as in aptasensors have long-term stability and can be easily synthesized for later use. In the case of food pathogens, some bacteria can be detected directly from the food sample but also to identify the target bacteria in samples within 35 min. In addition, some aptamers or aptasensors showed advantages such as rapidity, simplicity, and efficiency, which outweigh the limitations described for this technique [41].

Since there are currently no effective means of preventing infections mainly with Gram-negative bacteria, especially in hospital-care-associated infections (HCAIs), and blockage is often not detected until more serious symptoms arise, strategies for prophylaxis and a rapid early diagnosis are urgently required [107,108,109]. Such strategies would ideally permit the detection of nosocomial infections or initial urinary tract colonization and utilize samples that are easy to obtain, for example, for the detection of low levels of cells present in urine [41].

In some cases, it is difficult to establish a DNA-based approach for the detection and identification of some bacterium, like the *Vibrio* species, due to the high variation in genes but also in similarity in different species such as in *V. alginolyticus* and *V. parahaemolyticus*. Thus, in some studies, instead of using a specific gene for the selection of aptamers, the bacterium’s surface is used, since they it is very sensitive but also has a high specificity among other aquatic pathogens. Even though there is a lot of work to do, aptamer-based detection provides a novel sensitive approach for the detection of this type of bacterium which can have wide application prospects in the detection of microorganisms [102]. 

For some bacteria, aptamers against pathogens can not only supplement the deficiency of antibodies but can also enrich the aptamer database to develop efficient analytical regents and molecular diagnoses tools. For example, the selection of specific aptamers against *C. jejuni* and its application using aptamers are highly sensitive, so that much endeavor is encouraged to select DNA aptamers for *C. jejuni* recognition and signaling, acting as an important supplement of existing *C. jejuni* biosensor methods [65].

Aptamers are an attractive alternative to immunosensors and enzyme-based sensors for microorganisms monitoring. Those oligonucleotides are proving to be effective molecular recognition probes of high priority in food, drug quality, safety testing, disease diagnoses, etc. Aptamers are stable and easily synthetized and modified, and this is important in some cases such as bacteria detection in food samples, as described in the present review. The use of aptamers, instead of antibodies, is a promising tool because of the advantages previously outlined. This review showed various rapid, specific, robust, and highly sensitive method approaches to identify pathogenic bacteria using aptamers not only in foods but in nosocomial places. Even though there is a lot of work involved in implementing aptamer sensors or probes, the aptamer preparation systems are facilitating the development of more reliable aptamer-based commercial devices (i.e., biosensors). Thus, it is important to emphasize the use of combinatorial techniques in pathogen detection even in foodborne or nosocomial infections. Intriguingly, all methods discussed can be used for the detection of other targets by substituting the aptamer. Aptamer-conjugated nanoparticles have also shown a high preference, given the role of nanoparticles in the stabilization of the aptamer and the provision of additional affinity with dissociation [110].

Identifying bacterial pathogens at genus, species, and strain levels is indispensable in supporting appropriate diagnoses and treatment, assessing the disease burden, tracking sources, performing traceback investigations, and disclosing changes in the frequency of phylogenetic groups in human/animal disease and environmental niches. Concerning some bacteria with a poor degree of differentiation such as the one used in *Shigella* identification and serotyping, remains a daunting task, especially in developing countries [111].

This work describes the advances in the technology of aptamers, with the purpose of providing knowledge to develop new aptamers to diagnose and treat different microorganisms. The field of study regarding aptamers has great potential, so it is expected that in the future, when they replace the use of antibodies, they can be routinely used in the diagnoses and treatment of different pathologies caused by microorganisms.

Since rapid detection and identification of the causal pathogen of the disease is crucial for public health, this review aims to provide information that can improve the diagnosis and treatment of various diseases caused by different pathogens.

Taken together, as the aptamer field progresses, we expect that the aptamer-based analytical tools will become more accessible compared to antibodies in the areas of food-safety monitoring and disease diagnosis. 

## Figures and Tables

**Table 1:** DNA aptamer applications for Gram-negative pathogens’ detection.

Microorganism	Pathology	Application/Binding Validation Method	Aptamer Name/Target	Kd	Selection Method	Detection Limit	Ref.
*A. baumannii*	Nosocomial infections	Diagnostic tool/Not reported but tested in a previous work	Dual aptamer/Whole cell	6.8 ± 1.9 nM	SELEX	1 × 10^8^ CFU/mL	[24]
		Inhibition of infection in mice/murine model	Gold nanoparticle-DNA aptamer/Whole cell	4.7 μM	NR	67% of inhibition	[25]
		Diagnostic tool/RT-PCR and flow cytometry	A01/Whole cell	NR	SELEX	NR	[26]
*C. jejuni*	Gastroenteritis	Diagnostic tool/Enumeration with direct plating	ONS-23 Whole cell	292.8 ± 53.1 nM	SELEX	10^8^–10^9^ Cells	[27]
		Diagnostic tool/ELISA	Whole cell			100 CFU/mL	[28]
		Detection in milk/USA-FSIS	ONS-23TA/Whole cell	292.8 nM	SELEX	3 log CFU/g of sample	[29]
*E. coli* O157:H7	Gastroenteritis	Diagnostic tool/SEM	Aptamer/Whole cell	10.30 nM	SELEX	1.46 × 10^3^ CFU/mL	[30]
*E. coli 8739*		Diagnostic tool/Fluorescence microscopy	EcO 4R/Outer membrane proteins	1.65 nM	Competitive FRET	30 CFU/mL	[31]
*E. coli* NSM59		Diagnostic tool/Flow cytometry	EcA5-27/Whole cell	110 nM	SELEX	NR	[32]
*F. tularensis* subspecies *japónica* bacterial antigen	Tuleremia	Diagnostic tool/ALISA and dot blot analysis	Anti tuleremia aptamer/Killed bacteria	NR	SELEX	1.7 × 10^3^ bacteria/mL	[33,34]
*F. tularensis*	Food contamination	Bacteria detection in lettuce and soi/Real-time PCR analysis	Aptamer/Whole cell	NR	SELEX	1 × 10^8^ cells/mL	[35]
*K. pneumoniae*	Pneumonia	Diagnostic tool/Flow cytometry	Aptamer biotin-Ag-XK10/KPC-2	0.81 nM	SELEX	10^2^ CFU	[36]
		Diagnostic tool/Raman analysis	Aptamer KPBA1/DNA/Whole cell	NR	SELEX	3.4 × 10^3^ CFU/mL	[37]
*N.* *meningitidis*	Meningitidis	Diagnostic tool/Fluorescence-activated cell sorting	Aptamer K3 and K4/Whole cell	28.3 pM and 39.1 pM	SELEX	200 and 100 CFU/mL	[38]
*P. mirabilis*	Urinary tract infection	Diagnostic tool/Not specified	PmA109/cells pre-treated with proteinase K to degrade surface proteins	3.5 nM	SELEX	1 × 10^7^ CFU	[39]
*P. aeruginosa*	Infections, pneumonia	Diagnostic tool/Crystal violet assay, SEM, and confocal imaging	PmA2G02/Whole cell	NR	SELEX	No specified	[40]
		Pathogen determination in milk/TaqMan real-time PCR assays, multiplex recombinant polymerase amplification, and portable bacteria-capturing chip	DNA-SiC QDs/Whole cell	4.1 nM	Fluorescent aptasensor	526 CFU/mL	[41]
		Diagnostic tool/FISH assay	Aptamer F23/Inactivated cell	57.63 ± 11.64 nM	SELEX	1 × 10^7^ CFU/mL	[42]
			Aptamer/Whole cell	15.16 ± 3.62 nM.	SELEX	9 CFU/mL	[43]
		Diagnostic tool/Fluorescence assay	Aptamer/Whole cell	17.27 nM	Fluorometric aptamer probe	1 CFU/mL	[44]
*S. sonnei*	Diarrheal disease	Detection/Enzyme-linked aptamer sedimentation assay (ELAA)	ASA2/Whole cell	NR	SELEX	10^9^ CFU	[45]
		Diagnostic tool/Flow cytometry	Sp1 and Sp20/Whole cell	5.980 ± 0.835 nM and 14.32 ± 2.19 nM	SELEX	30 CFU/mL	[46]
*S. dysenteriae*	Salmonellosis	Diagnostic tool/Fluorescence assay	S1/Whole cell	23.47 ± 2.48 nM	SELEX	50 CFU/mL	[47]
			Aptamer/Whole cell	52.211.4 nM	SELEX	5.75 nM	[48]
*S. enterica* serovar Typhi	Systemic infection/Enteric fever	Diagnostic tool/Testing Vi antigen in urine and sera specimens	MoS_2_-rGO nanocomposite/Vi polysaccharide antigen	638.6 nM	SELEX	100 pg mL^−1^	[49]
*S. typhimurium*	Diarrheal diseases	Diagnostic tool/Electrophoretic mobility shift analysis	Aptamer 33/Outer-membrane proteins and LPS	NR	SELEX	<10^1^ CFU/g	[50]
*S. enteriditis*	Enteritis	Detection/ELAA	crn-1 and crn-2/Whole cell	0.971 µM and 0.309 µM	SELEX	1000 CFU/mL	[51]
*S.* O8	Salmonellosis	Diagnostic tool/aptamer-linked immobilized sorbent assay	B10/Whole cell	32.4 nM	SELEX	NR	[52]
*S. Typhimurium*	Salmonellosis	Diagnostic tool/Culture plating and PCR	Aptamer B5/Whole bacteria	58.5 nM	SELEX	80 CFU/mL	[49,53,54]
*V. parahaemolyticus*	Gastroenteritis	Diagnostic tool/Flow cytometry	SiO2@Au apt 1/Whole cell	16.88 ± 1.92 nM	SELEX	10^8^ CFU/mL	[55,56]
*V. alginolyticus*	Gastroenteritis	Detection/Flow cytometry	Aptamer VA2 and VA8/Whole cell	14.32 ± 4.26 nM and 90.00 ± 13.51 nM	SELEX	10^8^ CFU/mL	[57]
		Diagnostic tool/PCR	Aptamer P1:P3 pool/Whole cell	27.5 ± 9.2 nM	SELEX	100 cells/mL	[58]
		Diagnostic tool/ELISA	Vapt2/Whole cell	26.8 ± 5.3 nM	SELEX	10 to 10^7^ CFU/mL	[59]
*V. vulnificus*	Infection	Diagnostic tool/Deep sequencing	Vapt2/Whole cell	26.8 ± 5.3 nMnM	SELEX-PCR	8 − 2 × 10^8^ CFU/mL	[60]

NR: not reported.

## Data Availability

The data presented in this study are available on request from the corresponding author. The data are not publicly available because of privacy restrictions.
